# Cocaine-Seeking Behavior Induced by Orexin A Administration in the Posterior Paraventricular Nucleus of the Thalamus Is Not Long-Lasting: Neuroadaptation of the Orexin System During Cocaine Abstinence

**DOI:** 10.3389/fnbeh.2021.620868

**Published:** 2021-02-23

**Authors:** Alessandra Matzeu, Rémi Martin-Fardon

**Affiliations:** Department of Molecular Medicine, The Scripps Research Institute, La Jolla, CA, United States

**Keywords:** orexin, cocaine, pPVT, abstinence, OrxRs

## Abstract

Hypothalamic orexin (Orx) projections to the paraventricular nucleus of the thalamus (PVT) have received growing interest because of their role in drug-seeking behavior. Using an established model of cocaine dependence (i.e., long access [LgA] to cocaine), we previously showed that OrxA injections in the posterior PVT (pPVT) reinstated extinguished cocaine-seeking behavior in rats after an intermediate period of abstinence (2–3 weeks). Considering the long-lasting nature of drug-seeking behavior, the present study examined whether the priming effect of intra-pPVT OrxA administration was preserved after a period of protracted abstinence (4–5 weeks) in rats that self-administered cocaine under LgA conditions. Furthermore, to better understand whether a history of cocaine dependence affects the Orx system—particularly the hypothalamic Orx↔pPVT connection—the number of Orx-expressing cells in the lateral hypothalamus (LH), dorsomedial hypothalamus (DMH), and perifornical area (PFA) and number of orexin receptor 1 (OrxR1)- and OrxR2-expressing cells in the pPVT were quantified. Orexin A administration in the pPVT induced cocaine-seeking behavior after intermediate abstinence, as reported previously. At protracted abstinence, however, the priming effect of OrxA was absent. A higher number of cells that expressed Orx was observed in the LH/DMH/PFA at both intermediate and protracted abstinence. In the pPVT, the number of OrxR2-expressing cells was significantly higher only at intermediate abstinence, with no changes in the number of OrxR1-expressing cells. These data build on our previous findings that the hypothalamic Orx↔pPVT connection is strongly recruited shortly after cocaine abstinence and demonstrate that the priming effect of OrxA is not long lasting. Furthermore, these findings suggest that throughout abstinence, the Orx↔pPVT connection undergoes neuroadaptive changes, reflected by alterations of the number of OrxR2-expressing cells in the pPVT.

## Introduction

The paraventricular nucleus of the thalamus (PVT) plays a major role in regulating arousal, attention, awareness states, food consumption, and energy balance (Bentivoglio et al., 1991; Groenewegen and Berendse, 1994; Van der Werf et al., 2002; Colavito et al., 2015; Kirouac, 2015). The PVT has been consistently shown to be activated during periods of arousal and stressful conditions (Peng et al., 1995; Bhatnagar and Dallman, 1998; Novak and Nunez, 1998; Bubser and Deutch, 1999; Novak et al., 2000; Otake et al., 2002). The PVT has attracted interest because of its connections with limbic and cortical structures that are part of the neurocircuitry that mediates drug-seeking behavior (Everitt et al., 2001; McFarland and Kalivas, 2001; Ito et al., 2002; Kalivas and Volkow, 2005; Belin and Everitt, 2008; Steketee and Kalivas, 2011). The PVT is selectively recruited during cocaine-seeking behavior that is induced by the presentation of cocaine-predictive stimuli (Matzeu et al., 2017), and its integrity is necessary for behavior that is motivated by the presentation of cocaine-predictive environmental stimuli (Matzeu et al., 2015). Some of the pivotal components of the neurocircuitry of addiction (Koob and Volkow, 2010) receive projections from the PVT (Kirouac, 2015), highlighting the potential importance of this thalamic nucleus in the regulation of compulsive drug seeking that characterizes addiction. The expression of orexin (Orx), also known as hypocretin, is restricted to a small group of neurons in the hypothalamus: lateral hypothalamus (LH), dorsomedial hypothalamus (DMH), and perifornical area (PFA; de Lecea et al., 1998; Peyron et al., 1998; Sakurai et al., 1998). Although Orx-containing neurons represent a relatively small proportion of cells, their projections are widely distributed throughout the brain (Peyron et al., 1998), thus explaining how they can play diverse roles in physiological functions, including energy homeostasis, arousal, sleep/wake cycles (Sutcliffe and de Lecea, 2000; Mieda and Yanagisawa, 2002; de Lecea, 2012), and reward function (e.g., drug-seeking behavior; Harris et al., 2005; Dayas et al., 2008; Martin-Fardon et al., 2010, 2016; Jupp et al., 2011; Sakurai and Mieda, 2011). Orexin neurons project to structures that control behavior that is motivated by drugs of abuse, such as septal nuclei, the central nucleus of the amygdala, the ventral tegmental area, the medial prefrontal cortex, the nucleus accumbens shell, and the PVT, especially its posterior part (pPVT; Peyron et al., 1998; Baldo et al., 2003; Kirouac et al., 2005; Hsu and Price, 2009). Importantly, OrxA in the pPVT has been directly implicated in cocaine-seeking behavior (Matzeu et al., 2016). A microinjection of OrxA in the pPVT reinstated (primed) extinguished cocaine-seeking behavior in animals that had a history of extended access to cocaine, an established animal model of cocaine dependence (Matzeu et al., 2016). Remaining unknown, however, are whether the priming effect of OrxA is long lasting and whether a history of cocaine dependence affects the Orx system, particularly the hypothalamic Orx↔pPVT connection.

Therefore, because of the remarkable long-lasting resistance to the extinction of cocaine-seeking behavior (Martin-Fardon et al., 2016) and strong recruitment of the Orx system (Martin-Fardon et al., 2016) and PVT (Matzeu et al., 2017) during cocaine-seeking behavior, the aim of the present study was to test the ability of microinjections of OrxA directly in the pPVT to reinstate extinguished cocaine-seeking behavior at 2–3 weeks of abstinence (i.e., intermediate abstinence) or 4–5 weeks of abstinence (protracted abstinence). These two time points were chosen based on previous reports from our group that contrasted long-term persistence of the motivating effects of cocaine-related stimuli vs. rapid extinction of the motivational effects of stimuli that were conditioned to a highly palatable food reward (Martin-Fardon and Weiss, 2017; Martin-Fardon et al., 2018). These time points correspond to the first and fourth cocaine-related stimulus presentations. Within the same time period after the last cocaine exposure, cocaine-related stimuli still induced the strong recovery of responding, whereas the presentation of highly palatable food reward-related stimuli did not (Martin-Fardon and Weiss, 2017; Martin-Fardon et al., 2018).

Behavioral specialization is observed among hypothalamic subregions, with the LH playing a role in the promotion (reinstatement) of drug seeking (e.g., Marchant et al., 2009) and the DMH/PFA playing a major role in the inhibition of this behavior (Marchant et al., 2012). A functional difference between the two subtypes of Orx receptors, OrxR1 and OrxR2, has been suggested (Aston-Jones et al., 2010). OrxR1 signaling is mainly involved in reward function, and OrxR2 signaling is mainly involved in arousal states. Consequently, to further understand whether a history of cocaine dependence affects the Orx system, which could explain the reinstating effect of OrxA when injected in the pPVT, a secondary aim of the present study was to quantify the number of Orx-expressing cells in three subregions of the hypothalamus (LH, DMH, and PFA) that produce Orx and the number of OrxR1- and OrxR2-positive cells in the pPVT at intermediate and protracted abstinence.

## Materials and Methods

### Rats

Forty-three male Wistar rats (Charles River, Wilmington, MA, United States), weighing 200–225 g upon arrival in the laboratory, were housed two per cage in a temperature- and humidity-controlled vivarium on a reverse 12/12 h light/dark cycle with *ad libitum* access to food and water. The experiments were conducted during the dark phase. All of the procedures were conducted in strict adherence to the National Institutes of Health *Guide for the Care and Use of Laboratory Animals* and approved by the Institutional Animal Care and Use Committee of The Scripps Research Institute.

### Self-Administration and Extinction Training

**FIGURE 1:**
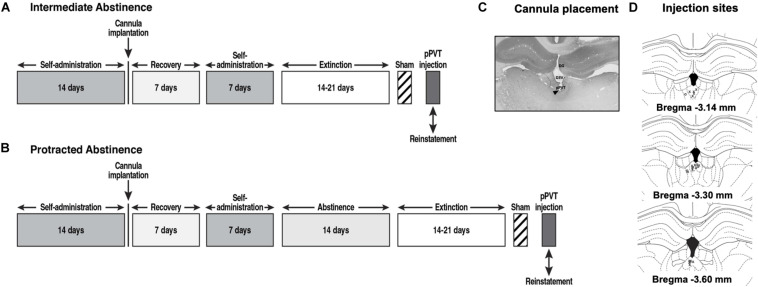
Behavioral procedure at **(A)** intermediate abstinence and **(B)** protracted abstinence. **(C)** Representative photomicrograph of the cannula placement. The black triangle indicates the injection site. DG, dentate gyrus; D3V, dorsal third ventricle. **(D)** Schematic distribution of injection site tracks in the pPVT (*x*, rats with correct cannula placements; o, rats with cannula misplacements).

Figure 1 Rats that were designated for cocaine self-administration were surgically prepared with jugular catheters 7–10 days before beginning cocaine self-administration training in 6 h/day (i.e., long-access [LgA]) sessions. Each session was initiated by the extension of two retractable levers into the operant conditioning chambers (29 cm × 24 cm × 19.5 cm; Med Associates, St. Albans, VT, United States). Responses at the active lever were reinforced on a fixed-ratio 1 (FR1) schedule by an intravenous (IV) infusion of cocaine (National Institute on Drug Abuse, Bethesda, MD, United States; 0.25 mg/0.1 ml/infusion) that was dissolved in 0.9% sodium chloride (Hospira, Lake Forest, IL, United States) and infused over 4 s. Each reinforced response was followed by a 20-s timeout (TO20) period that was signaled by the illumination of a cue light above the active lever. Responses at the inactive lever were recorded but had no scheduled consequences.

#### Cannulation

Fourteen days after beginning self-administration training, the rats that were designated for the reinstatement of cocaine seeking were implanted with a guide cannula (23-gage, 15 mm, Plastics One, Roanoke, VA, United States) that was aimed at the pPVT (anterior/posterior, −3.3 mm; medial/lateral, ±2.72 mm from bregma; dorsal/ventral, −2.96 mm from dura, 25° angle; Paxinos and Watson, 1997) and positioned 3.5 mm above the target injection point. After 7 days of recovery, the animals resumed self-administration training for an additional 7 days.

#### Extinction

The rats were divided into two subgroups with similar cocaine intake during self-administration training and tested at intermediate or protracted abstinence.

#### Intermediate Abstinence

Immediately following the completion of 21 daily self-administration sessions, the rats that were to be tested following intermediate abstinence underwent extinction training for 14–21 days (until the extinction criterion of ≤10 responses over three consecutive sessions was reached; Matzeu et al., 2016).

#### Protracted Abstinence

After the completion of 21 daily self-administration sessions, the rats that were to be tested following protracted abstinence were left undisturbed (with the exception of daily handling) in the vivarium for 14 days and then underwent extinction training for 14–21 days (until the extinction criterion of ≤10 responses over three consecutive sessions was reached), similar to intermediate abstinence rats. All of the extinction sessions lasted 2 h and began with extension of the levers into the operant chambers, with the same schedule of self-administration but without reward (cocaine) delivery.

### Intra-PVT Microinjections

Figure 1 Following the last day of extinction training, the rats that were designated for intra-pPVT OrxA prime-induced reinstatement received a sham injection (Sham) for habituation to the microinjection procedure. Twenty-four hours later, they received an intra-pPVT microinjection of 0.5 μg OrxA (Matzeu et al., 2016; American Peptide, Sunnyvale, CA, United States) in 0.9% sodium chloride (Hospira, Lake Forest, IL, United States) or vehicle (i.e., 0.9% sodium chloride; Matzeu et al., 2016). The microinjections in the pPVT were performed using a microinfusion pump (Harvard 22 Syringe Pump, Holliston, MA, United States) and injectors that extended 3.5 mm beyond the guide cannula. The injections were performed at a flow rate of 0.5 μl/min over 1 min. The injectors were left in place for an additional minute to allow diffusion away from the injector tip. Following the injections, the rats were returned to their home cages for 2 min and then placed in the operant chambers under extinction conditions for 2 h. After the test, the rats were euthanized by CO_2_ inhalation, and their brains were collected and snap frozen. The brains were sectioned coronally (40 μm) on a cryostat at −20°C, and injection tracks were verified (Figures 1C,D). Only rats with cannula placements that were located in the appropriate brain region were included in the data analysis.

### Immunohistochemistry

In parallel with the behavioral experiments, a separate subgroup of rats was designated for histology. Following the last day of extinction training (corresponding to the time at which the OrxA-induced reinstatement tests should have occurred) the rats (*n* = 5 for intermediate abstinence, *n* = 5 for protracted abstinence) were deeply anesthetized and transcardially perfused with cold 4% paraformaldehyde in 0.1 mM sodium tetraborate, pH 9.5. Brains were removed, postfixed in 4% paraformaldehyde overnight, and stored in 30% (w/v) sucrose, 0.1% (w/v) sodium azide, and potassium phosphate-buffered saline (KPBS) solution. The brains were sectioned coronally (40 μm) on a cryostat at −20°C and collected in a one-in-six series of adjacent sections. One section was then processed for OrxA immunodetection in the LH, DMH, and PFA, and the other two sections were processed for OrxR1 or OrxR2 detection in the pPVT. Briefly, coronal sections were blocked for 90 min using 5% normal donkey serum, 0.1% bovine serum albumin (BSA), and 0.3% Triton-X in PBS, followed by incubation for 48 h at room temperature with anti-OrxA antibody (1:15000, goat, Santa Cruz Biotechnology, Dallas, TX, United States), anti-OrxR1 (1:500, rabbit, Alamone Labs, Jerusalem, Israel), or anti-OrxR2 (1:500, rabbit, Alamone Labs, Jerusalem, Israel). The tissue sections were then incubated with ImmPress reagent with secondary antibodies for 90 min (anti-goat or anti-rabbit IgG, Vector Laboratories, Burlingame, CA, United States). OrxA, OrxR1, and OrxR2 immunostaining was visualized using DAB (Vectors Laboratories, Burlingame, CA, United States). Controls for antibody specificity were performed for all of the experiments by omitting the primary antibodies. This procedure was repeated for the secondary antibodies. OrxA-positive (Orx^+^) cells were counted within sections that incorporated the LH, DMH, and PFA (typical range: −2.40 and −3.48 from bregma). OrxR1^+^ and OrxR2^+^ cells were counted within sections that included the pPVT (typical range: −2.80 and −3.80 from bregma). As a control for basal Orx, OrxR1, and OrxR2 expression for all groups, brains from two age-matched naive groups of rats that were handled daily for 5 min but were not exposed to the behavioral chambers (*n* = 5 for intermediate abstinence, *n* = 5 for protracted abstinence) were also processed for immunohistochemistry. The brain tissues were then processed as above for the other animals. Although investigating how OrxA-pPVT prime-induced reinstatement causes additional changes in the Orx/OrxRs system could be of interest, the rationale for processing the tissue for immunocytochemistry was to evaluate the state of the Orx/OrxR system at the time when the rats were tested with a microinjection of OrxA in the pPVT (i.e., 24 h after the last extinction session).

### Statistical Analysis

The acquisition of cocaine self-administration was analyzed using two-way repeated-measures analysis of variance (ANOVA), with time (sessions) and lever (active vs. inactive) as factors. Reinstatement was analyzed using three-way ANOVA, with treatment (reinstatement conditions: extinction vs. sham vs. vehicle vs. OrxA), abstinence (intermediate vs. protracted), and lever (active vs. inactive) as factors. The number of Orx^+^, OrxR1^+^, and OrxR2^+^ cells was analyzed separately using two-way ANOVAs, with abstinence (intermediate vs. protracted) and group (naive vs. cocaine) as factors. Significant main effects or interactions were followed by the Tukey *post hoc* test. Pearson’s *r* correlation coefficients were calculated to establish linear dependence between the number of cocaine infusions that were earned during the last self-administration session and the number of Orx^+^, OrxR1^+^, and OrxR2^+^ cells. All of the results are expressed as mean ± SEM. Values of *p* < 0.05 were considered statistically significant. The statistical analysis was performed using GraphPad Prism 8 software.

## Results

Three rats were lost because of cannula misplacement, thus reducing the number of animals to 40 (intermediate abstinence: OrxA-induced reinstatement, *n* = 10 LgA; immunohistochemistry, *n* = 5 naive and *n* = 5 LgA; protracted abstinence: OrxA-induced reinstatement, *n* = 10 LgA; immunohistochemistry, *n* = 5 naive and *n* = 5 LgA).

### Cocaine Self-Administration Training and Extinction

**FIGURE 2:**
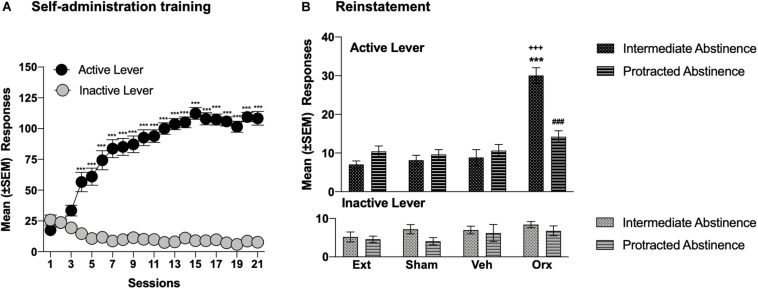
OrxA in the pPVT reinstated cocaine-seeking behavior at intermediate abstinence but not protracted abstinence. **(A)** Time course of cocaine self-administration over 21 days of training. ***p < 0.001, vs. first session and vs. inactive lever (Tukey *post hoc* test). *n* = 30. **(B)** An injection of OrxA (0.5 μg) in the pPVT induced cocaine-seeking behavior at intermediate abstinence but not protracted abstinence. ***p < 0.001, vs. extinction, sham, and vehicle; ^+++^*p* < 0.001, vs. inactive lever; ^###^*p* < 0.001, vs. OrxA at intermediate abstinence (Tukey *post hoc* test). *n* = 5–10. Ext, extinction; Sham, sham injection; Veh, vehicle; Orx, orexin A. The data are expressed as mean ± SEM.

Throughout the 21 days of self-administration training (6 h/day), the rats (*n* = 30) acquired cocaine self-administration (two-way ANOVA: session, *F*_20,1160_ = 29.45, *p* < 0.001; lever, *F*_1,58_ = 393.8, *p* < 0.001; session × lever interaction, *F*_20,1160_ = 57.31, *p* < 0.001; Figure 2A). The Tukey *post hoc* test confirmed that the rats increased their cocaine intake starting at session 4 vs. session 1 (*p* < 0.001) and vs. the inactive lever (*p* < 0.001). At the end of extinction training, the rats reached a comparable 3-day average (±SEM) number of responses between intermediate abstinence (7.0 ± 0.9 responses) and protracted abstinence (9.4 ± 1.1 responses; unpaired *t*-test, *t*_18_ = 1.625, *p* > 0.05). No differences were found in the number of sessions that were required to reach the extinction criterion between the two groups (intermediate abstinence: 18.0 ± 3 days, protracted abstinence: 19.0 ± 2 days; unpaired *t*-test, *t*_18_ = 0.6200, *p* > 0.05).

### Comparison of OrxA Priming Effects at Intermediate Abstinence vs. Protracted Abstinence

The injection of OrxA in the pPVT reinstated (primed) cocaine-seeking behavior at intermediate abstinence. At protracted abstinence, the priming effect of intra-pPVT OrxA was lost (Figure 2B). Indeed, at intermediate abstinence, rats that were injected with OrxA exhibited a significant increase in the number of responses at the active lever compared with extinction, compared with the sham injection, compared with the vehicle injection, and compared with the number of responses at the inactive lever (*p* < 0.001; Tukey *post hoc* test following three-way ANOVA: treatment, *F*_3,104_ = 24.30, *p* < 0.001; abstinence, *F*_1,104_ = 74.51, *p* < 0.001; lever, *F*_1,104_ = 7.112, *p* < 0.01; treatment × abstinence interaction, *F*_3,104_ = 12.66, *p* < 0.001; treatment × lever interaction, *F*_3,104_ = 8.98, *p* < 0.001; abstinence × lever interaction, *F*_1,104_ = 0.277, *p* > 0.05; treatment × abstinence × lever interaction, *F*_3,104_ = 8.541, *p* < 0.001; Figure 2B). Responses at the inactive lever remained low and unaffected.

### Number of Orx^+^ Cells in the LH, DMH, and PFA: Intermediate Abstinence vs. Protracted Abstinence

**FIGURE 3:**
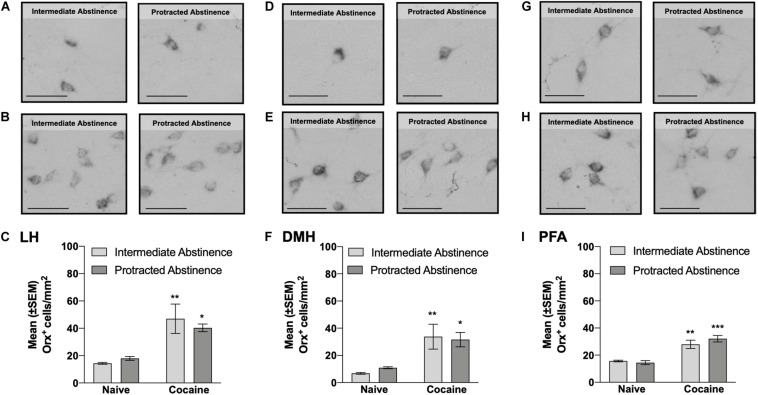
Number of Orx^+^ cells in the lateral hypothalamus (LH), dorsomedial hypothalamus (DMH), and perifornical area (PFA) measured at intermediate abstinence and protracted abstinence. **(A,B,D,E,G,H)** Typical photomicrographs of Orx immunostaining in naive rats **(A,D,G)** at the equivalent intermediate abstinence time point (left panels) and protracted abstinence time point (right panels) and in rats that self-administered cocaine LgA **(B,E,H)** at intermediate abstinence (left panels) and protracted abstinence (right panels). **(C,F,I)** A significantly higher number of Orx^+^ cells was observed in cocaine LgA rats vs. naive rats in the LH, DMH, and PFA at both intermediate abstinence and protracted abstinence. *p < 0.05, **p < 0.01, ***p < 0.001, vs. naive (Tukey *post hoc* test). Scale bars = 50 μm. The data are expressed as mean ± SEM.

Cocaine rats had a higher number of Orx^+^ cells compared with naive rats, with no differences between intermediate abstinence and protracted abstinence in the LH (*p* < 0.05; Tukey *post hoc* test following two-way ANOVA; abstinence [intermediate abstinence, protracted abstinence], *F*_1,16_ = 0.07, *p* > 0.05; group [naive, cocaine], *F*_1,__16_ = 23.96, *p* < 0.001; abstinence × group interaction, *F*_1,16_ = 0.83, *p* > 0.05; Figures 3A–C), DMH (*p* < 0.05; Tukey *post hoc* test following two-way ANOVA; abstinence [intermediate abstinence, protracted abstinence], *F*_1,16_ = 0.03, *p* > 0.05; group [naive, cocaine], *F*_1__,16_ = 20.14, *p* < 0.001; abstinence × group interaction, *F*_1,16_ = 0.35, *p* > 0.05; Figures 3D–F), and PFA (*p* < 0.01; Tukey *post hoc* test following two-way ANOVA; abstinence [intermediate abstinence, protracted abstinence], *F*_1,16_ = 0.50, *p* > 0.05; group [naive, cocaine], *F*_1__,16_ = 50.55, *p* < 0.001; abstinence × group interaction, *F*_1,16_ = 1.62, *p* > 0.05; Figures 3G–I). Correlation analysis did not reveal any significant relationship between the number of infusions that were earned during the last cocaine self-administration session and the number of Orx^+^ cells in the LH, DMH, and PFA (Table 1).

**TABLE 1:** Correlational analysis between the number of infusions earned during the last self-cocaine administration session and the number of Orx^+^, OrxR1^+^, and OrxR2^+^ cells.

		Intermediate abstinence	Protracted abstinence
Structure	Marker	*Correlation; significance*	*Correlation; significance*
LH		*r* = 0.20; *p* > 0.05	*r* = 0.26; *p* > 0.05
DMH	Orx^+^	*r* = 0.62; *p* > 0.05	*r* = 0.62; *p* > 0.05
PFA		*r* = 0.49; *p* > 0.05	*r* = 0.47; *p* > 0.05
pPVT	OrxR1^+^	*r* = 0.12; *p* > 0.05	*r* = 0.24; *p* > 0.05
pPVT	OrxR2^+^	*r* = 0.81; *p* > 0.05	*r* = 0.84; *p* > 0.05

### Number of OrxR1^+^ and OrxR2^+^ Cells in the pPVT: Intermediate Abstinence vs. Protracted Abstinence

**FIGURE 4:**
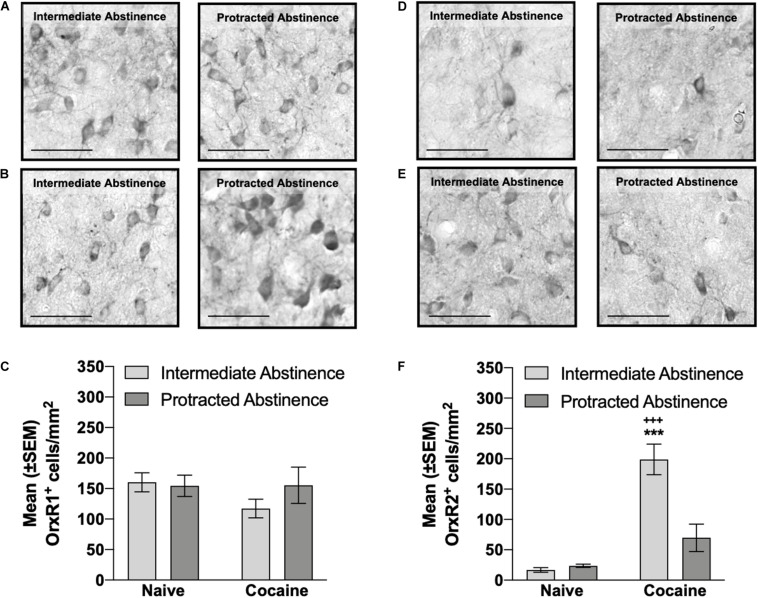
Number of OrxR1^+^ and OrxR2^+^ cells in the pPVT measured at intermediate abstinence and protracted abstinence. **(A,B)** Typical photomicrographs of OrxR1 immunostaining in naive rats **(A)** at the equivalent intermediate abstinence time point (left panel) and protracted abstinence time point (right panel) and in rats that self-administered cocaine with LgA **(B)** at intermediate abstinence (left panel) and protracted abstinence (right panel). **(C)** Rats that self-administered cocaine with LgA exhibited a similar number of OrxR1^+^ cells in the pPVT. **(D,E)** Typical photomicrographs of OrxR2 immunostaining in naive rats **(D)** at the equivalent intermediate abstinence time point (left panel) and protracted abstinence time point (right panel) and in rats that self-administered cocaine with LgA **(E)** at intermediate abstinence (left panel) and protracted abstinence (right panel). **(F)** Rats that self-administered cocaine with LgA had a significantly higher number of OrxR2^+^ cells in the pPVT at intermediate abstinence compared with rats that were exposed to cocaine at protracted abstinence and compared with naive rats. **^+++^***p* < 0.001, vs. cocaine protracted abstinence; ***p < 0.001, vs. naive (Tukey *post hoc* test). Scale bar = 50 μm. The data are expressed as mean ± SEM.

No differences in the number of pPVT OrxR1^+^ cells were observed at intermediate abstinence or protracted abstinence compared with naive (two-way ANOVA; abstinence [intermediate abstinence, protracted abstinence], *F*_1,16_ = 0.63, *p* > 0.05; group [naive, cocaine], *F*_1__,16_ = 1.06, *p* > 0.05; abstinence × group interaction, *F*_1,16_ = 1.15, *p* > 0.05; Figures 4A–C). The number of pPVT OrxR2^+^ cells was significantly higher at intermediate abstinence compared with naive and protracted abstinence, but no differences were detected at protracted abstinence compared with naive (*p* < 0.001; Tukey *post hoc* test following two-way ANOVA; abstinence [intermediate abstinence, protracted abstinence], *F*_1,16_ = 12.76, *p* < 0.05; group [naive, cocaine], *F*_1,16_ = 44.40, *p* < 0.001; abstinence × group interaction, *F*_1,16_ = 15.72, *p* < 0.01; Figures 4D–F). Correlation analysis did not reveal any significant relationship between the number of infusions that were earned during the last cocaine self-administration session and the number of OrxR1^+^ or OrxR2^+^cells in the pPVT (Table 1).

## Discussion

Because of the remarkable resistance to extinction and long-term persistence of cocaine-seeking behavior (Martin-Fardon et al., 2016), the ability of microinjections of OrxA directly in the pPVT to reinstate extinguished cocaine-seeking behavior at 2–3 weeks (i.e., intermediate abstinence) or 4–5 weeks (protracted abstinence) of abstinence was evaluated. In parallel, to further understand whether a history of cocaine dependence affects the Orx system and thus explain the reinstating effect of OrxA when injected in the pPVT at intermediate abstinence vs. protracted abstinence, we analyzed the number of Orx^+^ cells in the LH, DMH, and PFA and the number of OrxR1^+^ and OrxR2^+^ cells in the pPVT. We found a temporal change in OrxA’s priming effects, concomitant with temporal alterations of the number of OrxR2-expressing cells in the pPVT. Strong reinstatement was observed at intermediate abstinence, as reported previously (Matzeu et al., 2016), but no priming effects were detected at protracted abstinence. A higher number of Orx^+^ cells was observed in the LH/DMH/PFA at both intermediate and protracted abstinence. In the pPVT, the number of OrxR2^+^ cells was significantly higher at intermediate abstinence but not at protracted abstinence, with no changes in the number of OrxR1^+^ cells. These data indicate that shortly after cocaine abstinence, the hypothalamic Orx↔pPVT connection is strongly recruited. As the duration of abstinence is extended, this connection undergoes further neuroadaptive changes.

Consistent with previous studies (Matzeu et al., 2016, 2018), OrxA injections in the pPVT induced cocaine-seeking behavior at intermediate abstinence, supporting the importance of Orx projections to the PVT in the modulation of cocaine-seeking behavior, at least during the early stage of cocaine abstinence. A possible behavioral confound following the OrxA injection in the pPVT could be the close position of the pPVT to the third ventricle and thus the possibility that OrxA diffused to the ventricles and exerted non-specific actions at other brain regions beyond the pPVT. However, the accuracy of the injections (depicted in Figure 1), the lack of behavioral effects at protracted abstinence, and our earlier studies that used a similar approach (Matzeu et al., 2015, 2016, 2018; Matzeu and Martin-Fardon, 2020) strongly dispute this possibility. One may argue that microinjection of OrxA in the pPVT reinstated cocaine-seeking behavior at intermediate abstinence simply because of non-specific locomotor activation. However, the observation that responses at the inactive lever remained negligible and unaffected following the intra-pPVT OrxA injection (Figure 2B) suggests that the behavior was indeed specific (i.e., directed to the active lever) and not attributable to a general non-specific increase in locomotion. A tentative explanation for OrxA’s priming effects that were observed at intermediate abstinence may involve the mediation of arousal by the Orx system (Sakurai et al., 2010). In fact, the expectation of food reward was shown to activate neurons that contain OrxRs in the PVT (Choi et al., 2010). Most neurons in the PVT are sensitive to OrxA and OrxB, and the prefrontal cortex is an important target of Orx-activated PVT neurons (Ishibashi et al., 2005; Huang et al., 2006). The present results suggest that Orx inputs to the PVT might facilitate cortical activation that is linked to general arousal (Sato-Suzuki et al., 2002), which could explain the reinstatement of cocaine-seeking behavior. Moreover, OrxA administration in the PVT significantly increased dopamine levels in the nucleus accumbens (Choi et al., 2012), suggesting that the PVT is a key relay for Orx’s effects on the mesolimbic dopamine system and reward-seeking behavior.

Another mechanism by which Orx induces cocaine-seeking behavior could be related to the role of the PVT in mediating anxiety- and stress-like responses, which are known to precipitate drug-seeking behavior. The PVT sends projections to the dorsolateral bed nucleus of the stria terminalis and central nucleus of the amygdala. These structures contain neurons that densely express both dynorphin and corticotropin-releasing factor (CRF; Li and Kirouac, 2008). The peptides dynorphin and CRF are implicated in the expression of negative emotional states and stress responses (Davis, 1998; Heinrichs and Koob, 2004; Shirayama and Chaki, 2006; Davis et al., 2010). Both OrxA and OrxB injections in the PVT produced anxiety-like behavior in rats in the open field (Li et al., 2010a) and elevated plus maze (Li et al., 2010b), suggesting that Orx may act as a stressor and thus precipitate drug-seeking behavior. Moreover, knowing that Orx regulates the hypothalamic-pituitary-adrenal (HPA) axis (for review, see James et al., 2017) and that Orx levels in cerebrospinal fluid increase in patients with panic/anxiety disorder (Johnson et al., 2010, 2012), one possibility could be that Orx injections in the pPVT might activate the HPA axis and consequently increase corticosterone levels. However, to our knowledge, there is no evidence of a direct connection from the PVT to the paraventricular nucleus of the hypothalamus (e.g., Otake et al., 2002). Therefore, the hypothesis that microinjections of OrxA in the pPVT induce cocaine-seeking behavior through activation of the HPA axis requires further investigation. Surprisingly, however, the priming effect of OrxA was absent following a longer abstinence period (protracted abstinence). The reason for this temporal change in OrxA’s priming effect is unclear but may be attributable to differential neuroadaptive changes (e.g., pPVT OrxR2 expression), revealed by immunohistochemistry, that occurred as the duration of abstinence increased.

Although no correlation was found between the number Orx^+^ cells and the number of infusions that were earned during the last cocaine self-administration session, the persistent increase in the number of Orx^+^ cells during abstinence suggested that cocaine compromised the Orx system even after an extended cocaine-free period. This observation is consistent with earlier findings that described maladaptive recruitment of the Orx system by chronic cocaine. A recent study showed that intermittent access to cocaine (i.e., another animal model that induces an addiction-like state) results in a higher number of Orx-expressing neurons in the LH, and this increase persists for up to 150 days of abstinence (James et al., 2019). The same authors also observed greater Orx neuron activity in response to a cocaine-associated cue and greater efficacy of the OrxR1 antagonist SB334867 in reducing cocaine-seeking behavior (James et al., 2019). Rats with higher motivation for cocaine had a higher number of Orx cells in the LH, and knocking down these Orx cells reduced the motivation for cocaine (Pantazis et al., 2020), suggesting that the number of LH Orx cells may be a marker of addiction susceptibility. Orexin mRNA expression increased in the LH following chronic alcohol exposure (Lawrence et al., 2006), during withdrawal from cocaine (Zhou et al., 2008), in postmortem tissue from heroin addicts, and in mice that were exposed to chronic morphine (Thannickal et al., 2018). Glutamatergic inputs to Orx neurons increased following chronic cocaine exposure (Yeoh et al., 2012, 2019), further supporting our present findings of persistent changes in the Orx system following exposure to drugs of abuse.

The overall increase in the number Orx^+^ cells in the LH/DMH/PFA was somewhat unexpected when considering the reported functional dichotomy between hypothalamic subregions, in which Orx neurons in the LH participate in the regulation of reward processes, and Orx neurons in the DMH and PFA mediate responses to stressful events (Harris et al., 2005; Harris and Aston-Jones, 2006; Plaza-Zabala et al., 2010). The LH plays a central role in promoting and reinstating drug seeking, and the DMH/PFA plays a major role in inhibiting this behavior (Marchant et al., 2009, 2010, 2012). Corroborating these findings, concurrent intracranial self-stimulation of the DMH and LH decreased the reinforcing actions of self-stimulation of the LH (Porrino et al., 1983). Furthermore, administration of the inhibitory peptide cocaine- and amphetamine-regulated transcript in the DMH/PFA prevented the expression of extinction in a rat model of alcoholic beer seeking (Marchant et al., 2010). Considering evidence that the DMH/PFA regulates extinction and decreases LH activity (Porrino et al., 1983), activation of the DMH/PFA under physiological conditions may initiate the expression of extinction by inhibiting the LH (Porrino et al., 1983; Millan et al., 2011). Neuroplasticity that occurs during LgA cocaine self-administration and subsequent abstinence may prevent negative feedback from DMH/PFA neurons, such that LH neurons are no longer inhibited, reflected by a general upregulation of Orx in the LH/DMH/PFA. In agreement with this interpretation is a recent study that reported that intermittent access to fentanyl was associated with an increase in the number of Orx^+^ cells in the LH/DMH/PFA, suggesting the general overall adaptation of Orx-producing cells to drugs in general (Fragale et al., 2020). The exact mechanisms and adaptations that can explain such findings are unclear and will need to be studied further.

Neurons in the PVT express OrxR2 mRNA (Trivedi et al., 1998; Marcus et al., 2001), which is consistent with electrophysiological studies that reported that Orx depolarized and excited PVT neurons via OrxR2 (Ishibashi et al., 2005). The increase in OrxR2 levels at intermediate abstinence may reflect an elevation of OrxR2-mediated orexinergic transmission in the pPVT, which might be responsible for the ability of OrxA administration in the pPVT to induce cocaine-seeking behavior at intermediate abstinence only. PVT neurons are mostly glutamatergic (Hur and Zaborszky, 2005; Huang et al., 2006; Barroso-Chinea et al., 2007; Kolaj et al., 2014; Gupta et al., 2018). Thus, one possibility could be that a microinjection of OrxA in the pPVT increased glutamate release, which was previously shown by cellular recordings of pPVT slice preparations (Matzeu et al., 2018). Therefore, the activation of OrxR2 in the pPVT by a microinjection of OrxA might have increased glutamate release from pPVT neurons, which in turn may have been responsible for reinstating cocaine-seeking behavior at intermediate abstinence. This possibility requires further testing. As abstinence progresses, the persistent increase in OrxR2-mediated Orx transmission in the pPVT might induce a negative feedback mechanism that causes a reduction of OrxR2-expressing cells in the pPVT and consequently blocks the ability of OrxA to induce cocaine-seeking behavior at protracted abstinence. A persistent increase in the levels of OrxR2 but not OrxR1 has been reported following chronic injections of cocaine in the nucleus accumbens, but no changes were observed in the prefrontal cortex, ventral tegmental area, hypothalamus, or dorsal striatum (Zhang et al., 2007), suggesting that only specific receptor subtypes (e.g., OrxR2) within specific brain regions (e.g., the nucleus accumbens and pPVT) undergo neuroadaptations following chronic cocaine. This plasticity of the Orx/OrxR2 system may underlie relapse vulnerability after the cessation of drug use. This hypothesis is supported by previous findings from our group that showed that OrxR2 in the pPVT mediates the reinstating effect of OrxA in rats with a history of cocaine dependence at intermediate abstinence (Matzeu et al., 2016). The absence of a correlation between the number OrxR2^+^ cells and the number of infusions that were earned during the last cocaine self-administration session indicates that further studies are needed to define the precise mechanism by which such neuroadaptations occur during cocaine dependence and prolonged periods of abstinence.

In summary, the present study found that the discrete administration of OrxA in the pPVT elicited a priming effect that reinstated cocaine-seeking behavior in dependent animals at 2–3 weeks (intermediate abstinence) but not 4–5 weeks (protracted abstinence) of abstinence. The increase in Orx-expressing cells in the LH/DMH/PFA, together with the increase in OrxR2-expressing cells in the pPVT only at intermediate abstinence paralleled OrxA’s priming effect, suggesting that the hypothalamic Orx↔pPVT connection is strongly recruited shortly after cocaine abstinence. As the duration of abstinence increases, this connection undergoes further neuroadaptive changes. Remaining unknown are whether OrxA’s priming effect could be different at earlier abstinence (e.g., 24 h) vs. longer periods of abstinence (e.g., 6 months) and whether the Orx↔pPVT connection is differentially altered. Such findings may reveal valuable targets to mitigate the vulnerability to relapse that is associated with cocaine dependence that could be different as abstinence progresses.

## Data Availability Statement

The raw data supporting the conclusions of this article will be made available by the authors, without undue reservation.

## Ethics Statement

The animal study was reviewed and approved by all procedures were conducted in strict adherence to the National Institutes of Health Guide for the Care and Use of Laboratory Animals and approved by the Institutional Animal Care and Use Committee of The Scripps Research Institute.

## Author Contributions

AM and RM-F participated in the study concept and design. AM performed the experiments, undertook the statistical analysis, interpreted the findings, and drafted the manuscript. Both authors critically reviewed the content and approved the final version for publication.

## Conflict of Interest

The authors declare that the research was conducted in the absence of any commercial or financial relationships that could be construed as a potential conflict of interest.
